# Improved cycling performance and rate stability of ITO-compounded Li_2_MnSiO_4_ for lithium-ion batteries

**DOI:** 10.1039/c8ra00624e

**Published:** 2018-03-12

**Authors:** Jingya Liu, Yonghu Li, Shuai Yang, Jinjin Ai, Chunyan Lai, Qunjie Xu

**Affiliations:** Shanghai Key Laboratory of Materials Protection and Advanced Materials in Electric Power, Shanghai University of Electric Power Shanghai 200090 PR China laichunyan@shiep.edu.cn

## Abstract

Li_2_MnSiO_4_ compounded with indium tin oxide (ITO) was successfully synthesized through a sol–gel method. The structure and morphology characterization of Li_2_MnSiO_4_/ITO nanocomposite are demonstrated by XRD, SEM, TEM, EDS and XPS. Galvanostatic charge–discharge tests, EIS and CV are employed to examine the electrochemical performance of the composite. From those results, it could be observed that the electrochemical performance of Li_2_MnSiO_4_ cathode material has been significantly improved due to the introducing of indium tin oxide. The 3 wt% ITO-compounded sample displayed a discharge specific capacity around 141 mA h g^−1^ at 0.05C, 134.4 mA h g^−1^ at 0.1C, 132.9 mA h g^−1^ at 0.2C and 127.4 mA h g^−1^ at 0.5C in the first cycle, which is much higher than the pristine sample.

## Abstract 2

Li_2_MnSiO_4_ compounded with indium tin oxide (ITO) was successfully synthesized through a sol–gel method.

## Graphical abstract

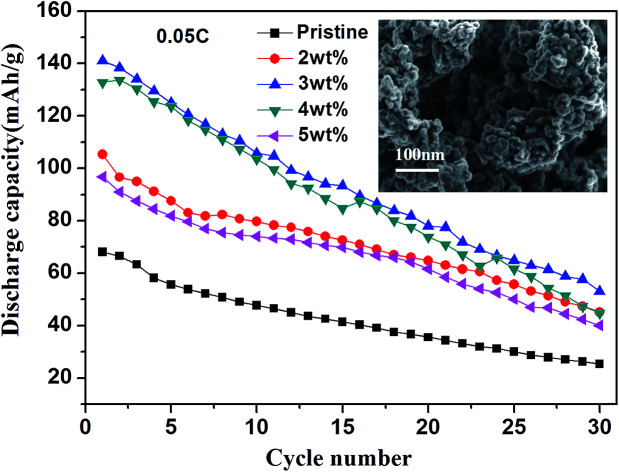

## 1. Introduction

With the development of the economy and the wide application of portable electronic devices, energy storage equipment is extensively utilized. Owing to their high energy density, long cycle life, being naturally abundant and environment-friendly and having low self-discharge, lithium-ion batteries dominate the main position of batteries. Polyanion compounds are a series of materials with tetrahedral or octahedral polyanion structure units (XO_*m*_)^*n*−^ (X = Si, P or As). Due to the strong X–O covalent bond in the middle of polyanion, this kind of cathode material can withstand over-charge or over-discharge and possess higher thermal stability than conventional cathode materials.^1,2^ Therefore, the lithium orthosilicate family, Li_2_MSiO_4_ (M = Fe, Mn, Co and Ni) have drawn widespread attention.

As for Li_2_FeSiO_4_, the potential window at 4.85 V (Fe^3+^/Fe^4+^)^3^ is too high to reach, the capacity was only confined to 166 mA h g^−1^.^4^ In comparison, it is quite possible to fully deintercalate two lithium ions from Li_2_MnSiO_4_ in principle, its theoretical specific capacity reaches 330 mA h g^−1^. However, low electronic conductivity and lithium-ion diffusivity hinder its commercial application. Since Li_2_MnSiO_4_ was synthesized for the first time in 2007 by Dominko,^5^ many researchers had made a great effort to enhance its electrochemical performances in the last decade.

Researches on computation and mechanism of Li_2_MnSiO_4_ had been conducted by many scientists. Li_2_MnSiO_4_ can be synthesized in different space groups, such as *Pmnb*, *Pmn*2_1_, *Pn* and P2_1_/*n*, and the orthorhombic *Pmn*2_1_ was reported most popularly due to its facile. Furthermore, density functional theory (DFT) calculations performed by M. M. Kalantarian *et al.*^6^ demonstrated that *Pmn*2_1_ was the best polymorph for using as a cathode material in lithium-ion batteries. Dompablo *et al.*^7^ quantify the inductive effect and calculate the linear dependence between voltage plateau of polyoxianionic compounds and the Mulliken X electronegativity.

Ion doping can improve the cycle stability due to its function of stabilizing the crystalline of Li_2_MnSiO_4_, which had been demonstrated by many researchers. For example, R. J. Gummow *et al.*^8^ synthesized magnesium-doped Li_2_MnSiO_4_ with *P*2_1_/*n* space group. Tingting Wu *et al.*^9^ prepared Ni-substituted Li_2_MnSiO_4_ by citric acid assisted sol–gel method. Meng Zhang *et al.*^10^ synthesized Li_2+*x*_MnSi_1−*x*_Al_*x*_O_4_/C nanoparticles by mixed solvothermal process.

Surface modifying restrains the growth of particle size during the annealing process and protect the dissolution of Li_2_MnSiO_4_ from HF in the electrolyte. As for Li_2_MnSiO_4_, many articles about coating have been published. Coating with carbon or metal oxide will help materials obtain superior cyclic performance, especially rate capability. Some metal oxide coatings are expected to neutralize the HF arise in the electrolyte.^11^ Jiangtao Zhu *et al.* had prepared MoO_2_ and carbon co-coated Li_2_MnSiO_4_,^12^ TiO_2_ and carbon co-modified Li_2_MnSiO_4_ (ref. 13) and ZnO coated Li_2_MnSiO_4_ (ref. 14) to improve its electrochemical performance.

Indium tin oxide (ITO, SnIn_2_O_3_) is an n-type transparent conductive semiconductor material.^15^ Because of excellent optical and electrical properties, it is widely applied in a variety of optoelectronic devices. While indium tin oxide was used as a compound material for Li_2_MnSiO_4_ has not been reported up to now. In this paper, we prepared Li_2_MnSiO_4_/ITO by sol–gel method, and compared physical and electrochemical characterization with the “standard” Li_2_MnSiO_4_. The relationship between the electrochemical performance and the ITO compounding on Li_2_MnSiO_4_ was explored.

## 2. Experimental

### 2.1 Synthesis of indium tin oxide (ITO)

The indium tin oxide (ITO) was synthesized by co-precipitation method. In order to obtain correct weight percent of ITO (In_2_O_3_ : SnO_2_ = 9 : 1), indium(iii) chloride and tin(iv) chloride are mixed in the beaker on the basis of calculation. Slight polyvinyl pyrrolidone was added to alleviate particles agglomeration. After dissolving completely with deionized water, NH_3_·H_2_O was dripped into the solution to adjust the pH range between 7 and 9. Magnetic stir thermostatically the solution at 60 °C for 2 h, and vacuum dry it to obtain a dry precursor. Then the pre-material is calcined at 400 °C for 5 h in atmosphere.

### 2.2 Synthesis of pristine and ITO-compounded Li_2_MnSiO_4_

To prepared the pristine and the ITO-compounded Li_2_MnSiO_4_, a stoichiometric amount (the molar ratio of Li : Mn is 2 : 1) of LiCH_3_COO·2H_2_O and Mn(CH_3_COO)_2_·4H_2_O are dissolved with deionized water in weighing bottles and citric acid is used as complexant to assist the synthesis of the composite. For modified materials, ITO (wt = 2, 3, 4, 5%) is added in and then ethanol is added to disperse Si(OC_2_H_5_)_4_ (TEOS). Magnetic stir thermostatically the solution at 65 °C for 10 h to form gel, and vacuum dry it for 20 h to obtain the precursor. Then the pre-material is calcined at 750 °C for 10 h in nitrogen atmosphere.

### 2.3 Structure and morphology characterization

The crystal structures of the synthesized materials were measured by D8 Advance X-ray diffractometer of Bruker. The samples were scanned from 10° to 80° at the rate of 6° min^−1^ with Cu Kα as the radiation source (*λ* = 0.15418 nm). The morphologies were recorded by a field emission scanning electron microscope (Hitachi, S-520) at 2 kV and transmission electron microscope (PHILIPS, FEGCM200).

### 2.4 Cells assemble and electrochemical performance characterization

The cells are assembled according to the following procedure: acetylene black as conductive carbon and PVDF as a binder are mixed with active material powder at the ratio of 1 : 1 : 8 by weight. After stirring for 2 hours, the slurry was coated firmly on aluminum foil and then evaporated for 8 hours. The electrolyte was 1 M LiPF_6_ in a mixture (1 : 1 : 1 volume ratio) of ethylene carbonate (EC), ethylmethyl carbonate (EMC), and dimethyl carbonate (DMC), meanwhile polypropylene microporous membrane was used as the separator. The CR2016 type coin cells were assembled in Ar-filled glove box. Galvanostatic charge and discharge tests were conducted on Land CT2001A battery test systems (Wuhan Jinnuo Electronics Co. Ltd., China) with the potential range between 1.5 V and 4.8 V at room temperature (293.15 K). Electrochemical impedance spectroscopy (EIS) and cyclic voltammetry (CV) measurements were carried out using an electrochemical workstation CHI 660E (Shanghai Chenhua, China).

## 3. Result and discussion

### 3.1 Structure and morphology of ITO-compounded Li_2_MnSiO_4_

**Fig. 1:**
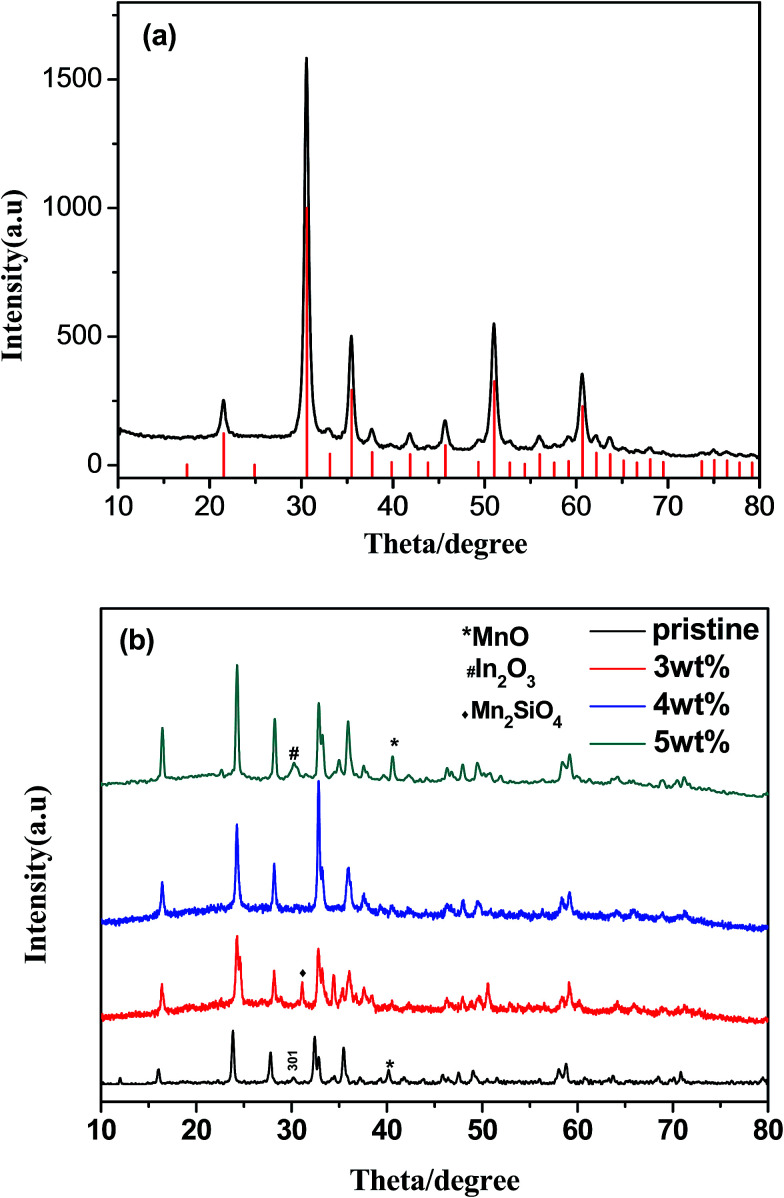
XRD pattern of ITO (a), pristine sample and ITO-modified Li_2_MnSiO_4_ (b).

Fig. 1a is the powder X-ray diffraction pattern of the synthesized ITO. All the characteristic peaks are indexed to In_2_O_3_ (JCPDS#71-2195) without any trace of tin or other tin compounds, which means that tin atoms have doped into In_2_O_3_ lattices.^16^Fig. 1b reveals the XRD patterns of pristine sample and ITO-compounded Li_2_MnSiO_4_ materials. Because the differences in formation energies of those phases are very small, the pristine sample was considered to be polymorphs mixture of *Pmn*2_1_ and *Pmnb* due to the characteristic *Pmnb* peak (301) at 30.5°.^17^ While, the synthesized composites Li_2_MnSiO_4_/ITO (*w* = 3, 4, 5%) were identified as orthorhombic crystal structure with a space group of *Pmn*2_1_, and the characteristic peaks are in good agreement with articles.^9,18–20^ Besides, the major characteristic peaks of modified materials are sharp, showing a good crystallinity. The results indicated that ITO adding will inhibit the formation of *Pmnb* in Li_2_MnSiO_4_. As impurities, MnO and Mn_2_SiO_4_ were allowed in some samples, which have been reported in previous literatures.^18,20^ There is no evidence of diffraction peaks for ITO in the XRD patterns of Li_2_MnSiO_4_/ITO (*w* = 3, 4%), may due to its low content.^14^ However, in the XRD patterns of Li_2_MnSiO_4_/ITO (*w* = 5%) sample, a diffraction peak at 30.58° could be assigned to the most strong peak of ITO (Fig. 1a) due to a higher content.

**Fig. 2:**
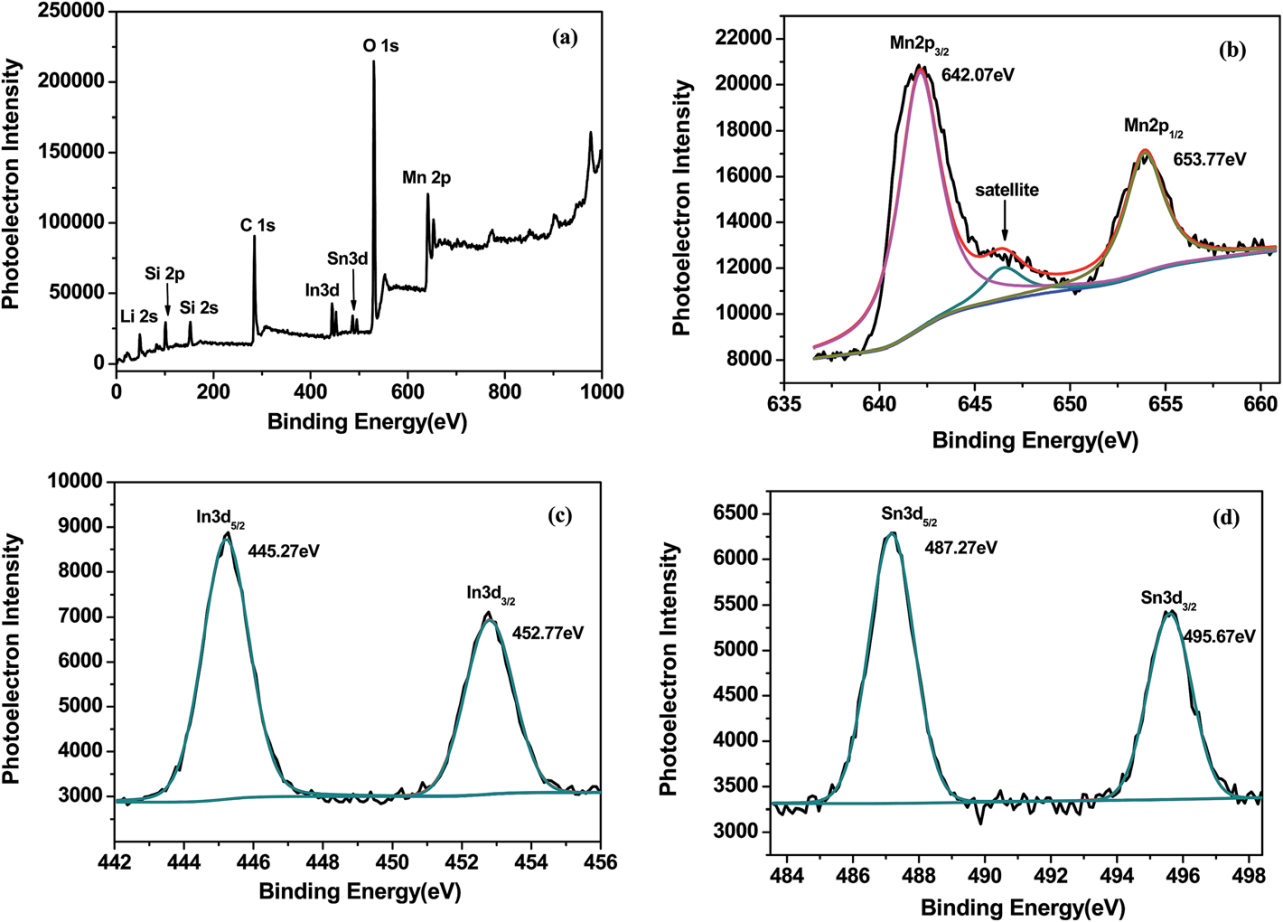
XPS wide spectrum of Li_2_MnSiO_4_/ITO (a) and enlarge figures of Mn 2p (b), In 3d (c) and Sn 3d (d) spectra of Li_2_MnSiO_4_/ITO.

The X-ray photoelectron spectroscopy was used to analyze the oxidation state of main elements in samples, and the survey spectrum was presented in Fig. 2a, the obtained binding energy (BE) had been calibrated by the BE of C 1s at 284.5 eV. The binding energy of Mn 2p_3/2_ (642.07 eV) and Mn 2p_1/2_ (653.77 eV) correspond to the divalent state of Mn.^21^ Besides, the shoulder satellite peak at 646.5 eV is a rigorous proof for Mn^2+^.^22^ In the narrow scan of In, the BE of 445.27 eV and 452.77 eV belong to In^3+^. Fig. 2d showed the Sn 3d XPS spectrum, where the peaks locate at 487.27 eV and 495.67 eV corresponding to Sn 3d_5/2_ and Sn 3d_3/2_ respectively. The peaks position demonstrated the quadrivalent state of Sn well.^15^

The sample was etched with Ar for 0 s, 5 s, 10 s and 15 s, the etched depth was 2 nm s^−1^. The Height CPS and atomic ratio of elements in Li_2_MnSiO_4_/ITO with Ar etched were presented in Table 1. The Height CPS represents photoelectron intensity. Although the sample was etched for different time, the atomic ratio of metal elements has not varied in large range. The ratios of In and Sn maintain in 0.63% and 0.3% indicate that In and Sn appear not only on the surface but also in the interior of Li_2_MnSiO_4_/ITO, and indium tin oxide (ITO, SnIn_2_O_3_) compounded with Li_2_MnSiO_4_ uniformly.

**Table 1:** Height CPS and atomic ratio of elements in Li_2_MnSiO_4_/ITO and Li_2_MnSiO_4_/ITO with Ar etched

Elements	Height CPS (0 s)	Atomic% (0 s)	Height CPS (5 s)	Atomic% (5 s)	Height CPS (10 s)	Atomic% (10 s)	Height CPS (15 s)	Atomic% (15 s)
O 1s	178 607.9	41.05	195 644.4	41.82	179 092	40.17	164 791.93	39.89
C 1s	67 465.16	42.96	70 640.1	40.91	74 323.89	44.25	73 470.38	45.86
Mn 2p	56 662.64	5.24	63 922.73	5.71	58 007.44	5.63	50 309.82	4.82
Si 2p	17 520.89	9.82	19 534.17	10.59	17 317.78	9.03	15 454.95	8.54
In 3d	21 943.58	0.63	23 550.45	0.65	21 338.04	0.63	19 352.59	0.62
Sn 3d	10 885.4	0.31	12 789.43	0.31	11 242.63	0.3	10 206.4	0.27

**Fig. 3:**
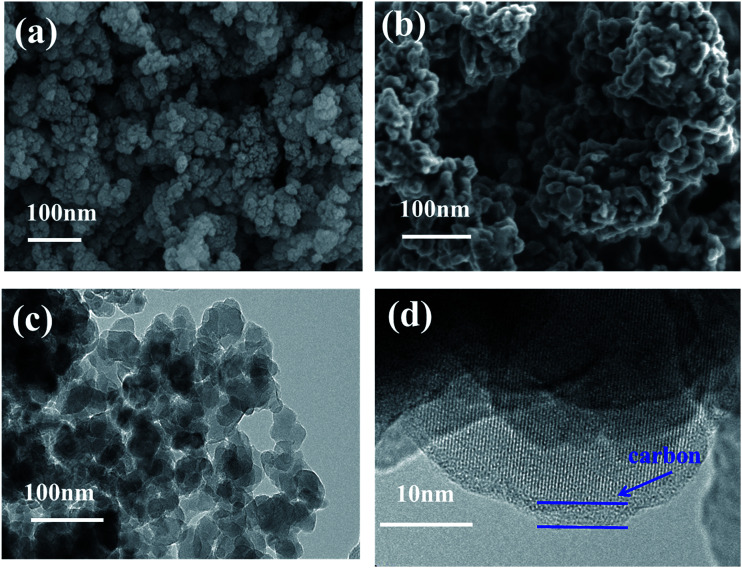
SEM images of Li_2_MnSiO_4_ (a) and 3 wt% ITO-modified Li_2_MnSiO_4_ (b) and TEM images of 3 wt% ITO-modified Li_2_MnSiO_4_ (c and d).

The morphology of Li_2_MnSiO_4_ and Li_2_MnSiO_4_/ITO (*w* = 3%) composites were presented in Fig. 3. Fig. 3a exhibits the pristine sample composed of nanoscale particles, and Fig. 3b shows the modified material. Compared with the pristine, the surface morphology of modified sample did not change visibly after compounding with 3 wt% ITO, however, the particle size varying from 20 nm to 50 nm was smaller than the pristine. Due to the small particle size, great surface tension and high surface free energy, agglomeration phenomenon may easily occurs.^23^

Transmission electron microscopy pictures of Li_2_MnSiO_4_/ITO nanoparticles were shown in Fig. 3c and d. Due to the samples were prepared *via* citric acid assisted sol–gel method, a thin carbon layer can be observed in Fig. 3d.^24^ The particle agglomerated slightly, lattice fringe can be seen from HR-TEM image, which suggests that a good crystallization of Li_2_MnSiO_4_/ITO were synthesized *via* sol–gel method.

**Fig. 4:**
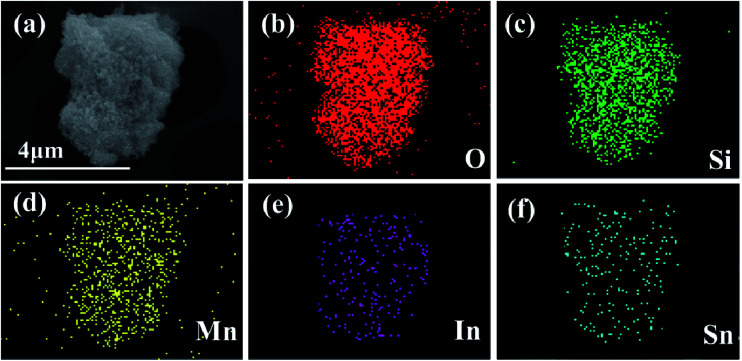
SEM images of 3 wt% ITO-modified Li_2_MnSiO_4_ (a) and corresponding elements distribution mapping of O, Si, Mn, In, Sn in the sample (b–f).

Fig. 4 exhibits the SEM image with the corresponding elemental mappings and Energy Dispersive Spectroscopy (EDS) images of 3 wt% ITO-compounded sample to demonstrate the uniform distribution of elements. Elements of Si, Mn, O, In, and Sn dispersed homogeneously on the surface of material. No other impurity elements are detected in the mapping image.

### 3.2 Electrochemical performance of ITO-compounded Li_2_MnSiO_4_

**Fig. 5:**
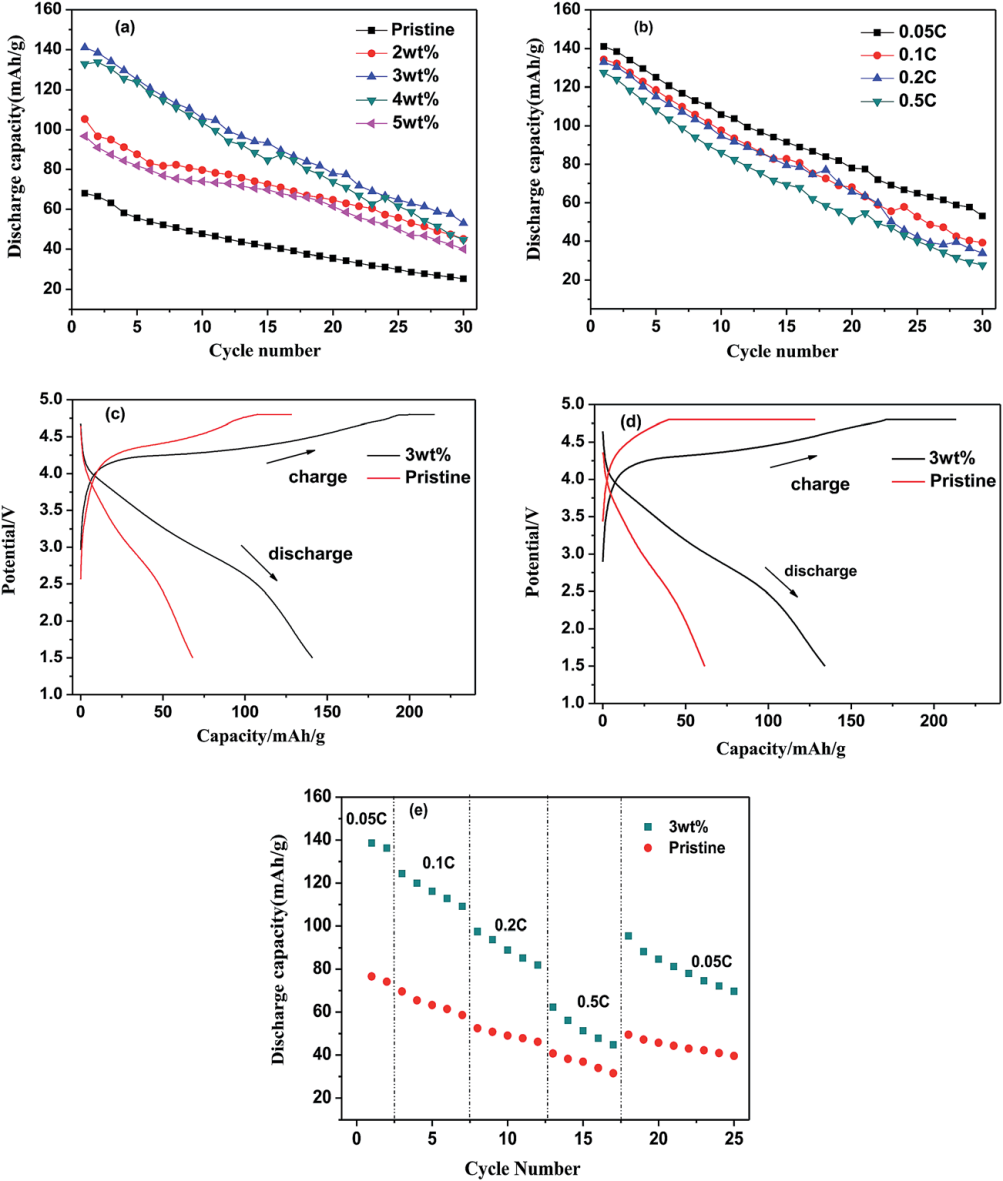
The discharge capacity curves of varied contents of ITO at 0.05C (a), the discharge capacity curves of 3 wt% ITO-compounded Li_2_MnSiO_4_ in different rate (b), the initial cycle discharge capacity of 3 wt% ITO-compounded Li_2_MnSiO_4_ at (c) 0.05C and (d) 0.1C, the rate capability of pristine and 3 wt% ITO-compounded Li_2_MnSiO4 (e).

Fig. 5 shows the charge/discharge profiles of LMS/ITO. Capacity of modified-LMS has promoted significantly compared with the pristine. It could be seen from Fig. 5a that the material compounded with 3 wt% ITO displays the best electrochemical performance. The Li_2_MnSiO_4_/ITO (*w* = 3%) sample delivered a discharge specific capacity around 141 mA h g^−1^ at 0.05C in the first cycle which is much higher than that of LMS (68.1 mA h g^−1^). It may be attributed to that ITO stabilize the structure of material and suppresses the amorphization of Li_2_MnSiO_4_ during the cycle. And the degradation in discharge capacity during cycles has been claimed to be due to the mixed valence state of Mn and the Jahn–Teller effect in LMS materials.^25^ While the decreased capacities of 4 wt% and 5 wt% samples reflect that there is an appropriate amount for compounding and excess amount of ITO will result in negative effect on the performance of the Li_2_MnSiO_4_/ITO. Excessive ITO may hinder the transportation of Li^+^, hence a large number of experiments were carried to pick out the optimal quantity.

ITO-introducing not only raises the specific capacity but also enhances the stability of Li_2_MnSiO_4_. Even at the rate of 0.1C, 0.2C and 0.5C in Fig. 5b, the initial discharge capacity reach 134.4 mA h g^−1^, 132.9 mA h g^−1^, 127.4 mA h g^−1^ separately. Compared with some reported results,^12,14^ the synthetic material in this research also obtain high rate discharge ability. For comparison, Fig. 5c and d present the initial cycle curves at 0.05C and 0.1C of pristine and 3 wt% ITO-modified samples. The discharge capacities of modified samples are obviously higher than those of pristine sample. In Fig. 5e, the LMS/ITO cathode shows better rate stability than the LMS cathode. It comes to a conclusion that the better electrochemical performance of LMS/ITO can be attributed to indium tin oxide.

**Fig. 6:**
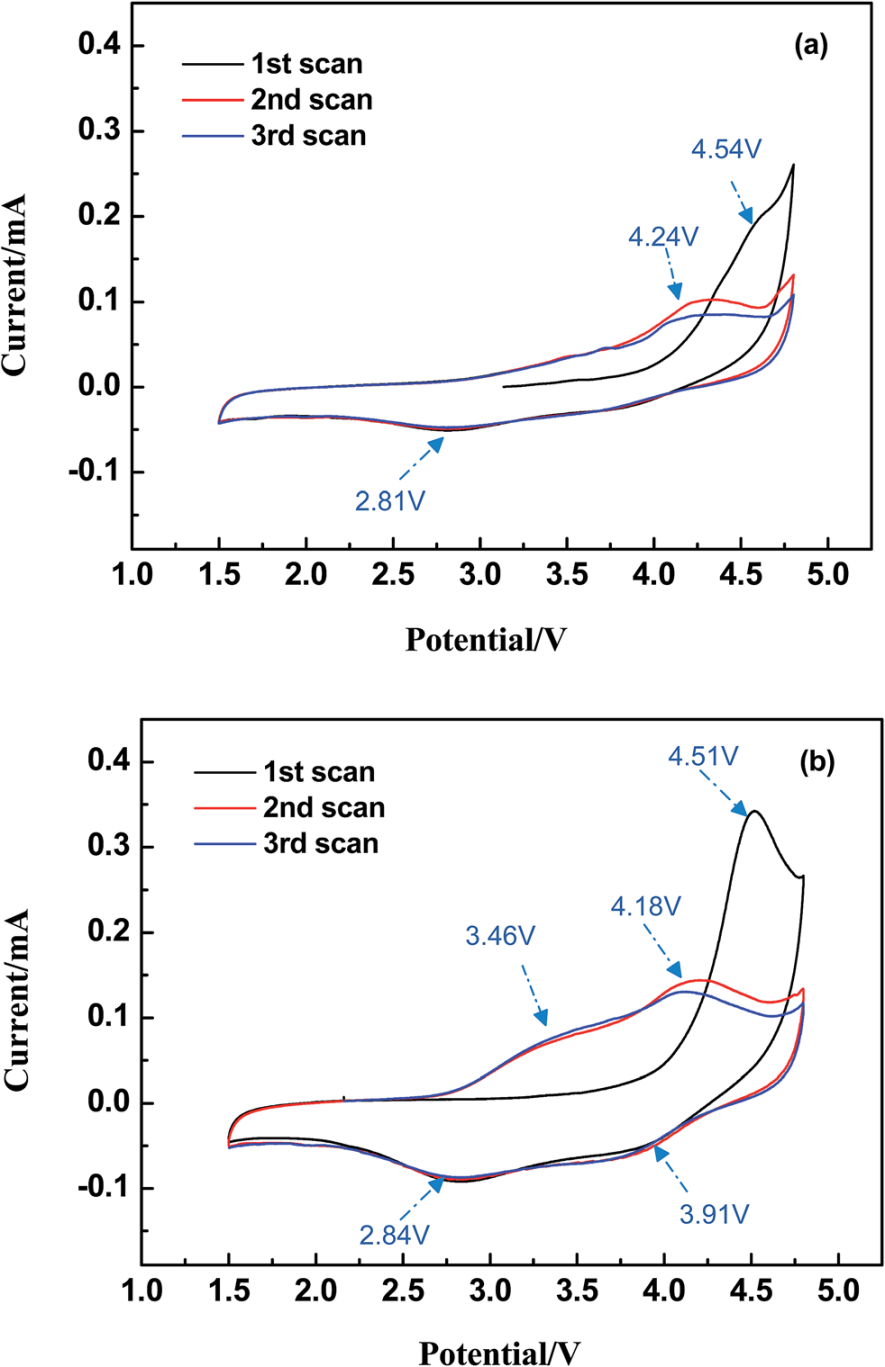
The cyclic voltammograms curves of Li_2_MnSiO_4_ (a) and Li_2_MnSiO_4_/ITO (b).

The cyclic voltammograms (CV) were measured at a scan rate of 0.1 mV s^−1^ ranging in potential windows from 1.5 V to 4.8 V. As it is depicted in Fig. 6, both LMS and LMS/ITO have different initial cycle with the following two CV curves, which illustrates that the structure of LMS and LMS/ITO may change to an amorphous state on the first charge–discharge process and a little part of LMS crystallites had decomposed.^26,27^ In the following cycles, LMS and LMS/ITO showed highly overlap curves, which mean better reversibility. In Fig. 6a, there is an oxidation peak around 4.24 V, corresponding to the extraction of lithium ion from Li_2_MnSiO_4_ cathode and a reduction peak at 2.81 V refers to the insertion of lithium ion.^27^ In Fig. 6b, the oxidation/reduction peaks at 4.18 V/3.91 V and 3.46 V/2.84 V respectively are ascribed to the Mn^3+^/Mn^4+^ and Mn^2+^/Mn^3+^ redox processes.^28^ Compared with the pristine, the oxidation peaks of LMS/ITO shifted to low potential and the reduction peaks moved higher and the oxidation/reduction current peaks of the LMS/ITO are larger. All above data illustrate that LMS/ITO has higher electrochemical activity than LMS, and the electrochemical reactions in LMS/ITO is more easily to occur. As for peaks area, LMS/ITO is larger than LMS, which reduces the polarization of electrode.

**Fig. 7:**
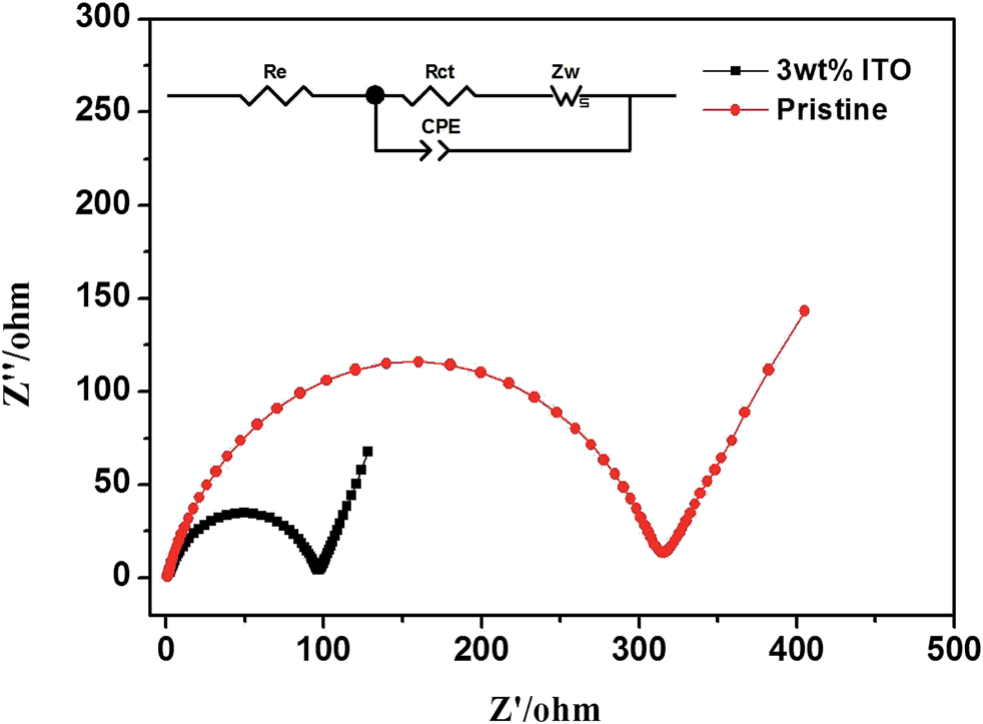
The EIS spectrum of pristine and ITO-modified samples and the equivalent circuit are inserted.

Fig. 7 exhibits the electrochemical impedance spectrum (EIS) of the samples, which is beneficial to analyze the kinetic properties. The equivalent circuit model, *R*_e_ and *R*_ct_ are calculated by Zview software. The diffusion coefficient is calculated as the following equations.^29^1
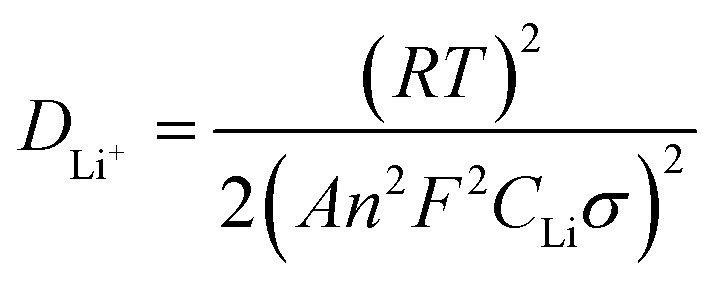

2 *Z*_re_ = *R*_e_ + *R*_ct_ + *σϖ*^−0.5^

where *R* is the gas constant (8.314 J mol^−1^ K^−1^), *T* is the room temperature (298.15 K), *A* is the surface area of the electrode, *F* is the Faraday's constant (96 485 C mol^−1^), *C* is the molar concentration of Li^+^ in materials and *σ* is Warburg factor, the slope of *Z*_w_*vs. ω*^−1/2^ and *ω* = 2π*f*. *R*_e_ is the ohmic resistance, representing the resistance of the electrolyte. *R*_ct_ is the charge transfer resistance of electrochemical reaction. The straight line at the low frequency corresponds to the Warburg impedance which is attributed to the diffusion of the Li-ion into the bulk of the electrode material.

Table 2 lists the calculation for both samples. Compared with the uncompounded material (306.9 Ω and 7.84 × 10^−17^ cm^2^ s^−1^), the modified sample with smaller *R*_ct_ (93.67 Ω) and larger *D*_Li^+^_ (3.67 × 10^−16^ cm^2^ s^−1^) has more efficient electron conductivity and lithium ion insertion/extraction.

**Table 2:** Parameters of impedance and calculated diffusion coefficient of lithium-ion

Samples	*R*_e_ (Ω)	*R*_ct_ (Ω)	*D*_Li^+^_ (cm^2^ s^−1^)
Li_2_MnSiO_4_	2.624	306.9	7.84 × 10^−17^
Li_2_MnSiO_4_/ITO	1.645	93.67	3.67 × 10^−16^

For root reason, as a dopant a modicum of Sn caused defects in the microstructure and then change the grain boundaries of ITO.^30^ The carrier concentration of the sample increases and the Hall mobility decreases with the ITO compounded, which lead to smaller resistivity.^16^ It indicates that the inclusion of ITO in the Li_2_MnSiO_4_ increase the Li-ion diffusion kinetics and offer lower charge transfer resistance, all of these are consistent with the charge/discharge performance of Li_2_MnSiO_4_/ITO.

## 4. Conclusion

Li_2_MnSiO_4_/ITO nanocomposites were successfully prepared *via* sol–gel method. The material compounded with 3 wt% ITO showed the best electrochemical performance. The initial discharge capacity achieved 141 mA h g^−1^, 134.4 mA h g^−1^, 132.9 mA h g^−1^ and 127.4 mA h g^−1^ separately at 0.05C, 0.1C, 0.2C and 0.5C. Compounding with ITO is an effective method to reduce the resistance and enhance the inherent conductivity, which lead to superior electrochemical performance.

## Conflicts of interest

There are no conflicts to declare.

## Supplementary Material
